# Pennsylvania State Dental Society

**Published:** 1885-01

**Authors:** Wm. H. Trueman


### PENNSYLVANIA STATE DENTAL SOCIETY.

SIXTEENTH ANNUAL MEETING, HELD AT WILKESBARRE, JULY 29, 30 AND 31, 1884.

REPORTED FOR THE INDEPENDENT PRACTITIONER BY WM. H. TRUEMAN, D.D.S.

(Concluded from page 688, Vol. V.)

### AFTERNOON SESSION OF WEDNESDAY, JULY 30.

The session was opened by Dr. Allen G. Bennett, of Philadelphia, who read a paper upon Operative Dentistry.

He suggested that in preparing a cavity, as far as possible those portions of the walls formed by the enamel should be made to conform to its lines of cleavage. Where this cannot be done, they should be beveled outward at an angle of from twenty-five to thirty-five degrees, almost its entire thickness; the softer the teeth the greater the necessity of this precaution. He thinks the neglect of this is often the cause of broken down margins around fillings. Although the enamel borders may seem strong, if the line of cleavage is undercut, as it may be in many cases where the walls are straight, the enamel is apt to give way at this point, and render the margin imperfect. He suggests that this could be studied by working upon a tooth that had been some time out of the mouth, and was perfectly dry, the lines of cleavage being more readily demonstrated than in a tooth in its normal position and condition. He recommended that the enamel edge should be carefully polished in all cases before commencing to fill.

He prefers grooves to retain the filling, only using retaining pits as a convenience in starting, and recommended the use of alkalies, antiseptics, or local anæsthetics in the cavity, where the condition of the tooth calls for them. In frail, sensitive teeth, he protects the dentine from contact of metallic fillings by a non-conductor. He thought much of the harshness complained of in cohesive gold was due to overheating in annealing, and overhammering, and that if care were used in annealing, and convex points with fine transverse serrations used in filling, the gold could be packed solidly and accurately, and uniform density with perfect welding be more certainly secured. Much has been said and written about moisture-tight fillings, but excavating with the oral fluids entirely excluded shows clearly that all the dentine is not "as dry as dust," and probably the moisture around a filling, in a soft tooth at least, may be entirely internal.

On the approximal surfaces of molars and bicuspids, he preferred to make a wide separation, and then restore the natural contour of the tooth. By this means he gets rid of weak edges, and removes the margins of the filling to a point easily inspected and kept clean. Contour is really separation made permanent; all the good of separation is obtained without its evils.

The weakest point of approximal fillings is always the cervical walls. This, he thought, is owing in a measure to the difficulty of properly preparing them. Quite frequently the engine cannot be used, and often when it is, it does not leave as smooth and level a surface as is desired. He has devised several instruments to more readily reach this point. They are somewhat like a hoe excavator, with a wide cutting edge, so shaped and bent at such an angle with the shaft that it is brought to bear upon the work very much as is the chisel of a carpenter's plane, and on this account it makes a smooth, clean cut, and can be made to follow the entire cervical border with one continuous sweep. The dentine at this point is usually soft, and requires a cutting tool with a thin edge brought to an acute angle. He exhibited several instruments, and they no doubt will accomplish the work well, and can be used effectively in a large proportion of cases. By modifying the cutting edge he finds them useful in beveling the enamel. He recommends using at this point soft gold when the cavity is quite accessible; in other cases prefers tin or amalgam, the latter for all deep cavities.

*Dr. Gerhart*—Suggested that when the rubber dam is on for a long time, especially when the hot air syringe is used and the cavity becomes perfectly dry, the tooth contracts, owing to the loss of moisture; the filling being put in while the tooth is in this condition, will not fit as accurately when the tooth returns to its normal state.

This gave rise to a long discussion, some contending that no change takes place; others that the tendency will be to make a more accurate fit between the tooth and the filling; and others again, that this change, though slight, is a source of danger, and the frequent cause of failures.

*Dr. William H. Trueman*—Related a case in which he had built up a tooth with the electric mallet, building the gold around the remaining portion of the tooth. Some months after, the root was split by violence, and had to be extracted. The gold and the portion of the tooth removed were firmly united, and after laying in the instrument case six or eight months there was no change; they were so dovetailed into each other that they could not be separated without fracture. They remained some twelve months more, almost forgotten, when they were again examined; the contraction was now so great that they fell apart. This demonstrates that when the dentine and enamel become perfectly dry there is a very perceptible contraction, but in this case it must have been about a year before it was noticeable. He did not think it possible for any material change to take place in the mouth during the short time a tooth would be covered by the dam, under any circumstances.

*Dr. Darby*—Thought the use of excessively cohesive gold, which is so apt to bridge and not pack down solidly at the walls, was more likely to be the cause of the trouble complained of than any contraction of the tooth, or change in the shape of the cavity. Except in special cases, he recommended a semi-cohesive gold as being more manageable.

*Dr. Wingate*—Referred to the old time methods by which good work was done without the rubber dam, and urged a return to the old practice in cases where it could be used to advantage.

*Dr. Pierce*—Spoke of gutta percha, recommending the pink in all cases where the filling was concealed. It contains less clay, and seems more durable than the white.

*Dr. Darby*—Mentioned the "Bevins" gutta percha, an article on the market some twenty years ago, but not now obtainable, as the best

3

he had ever known. There are several preparations that are good, but none equal to that.

*Dr. E. H. Neall*—Considered a good article of Hill's stopping to be the best filling for the approximal cavities in the teeth of children, until the twelfth or fifteenth year. Premium stopping, made by Johnson Bros., now sold by the S. S. White Co., he considered the best. He used it in small pieces, heating it on a metal plate, and carrying it quickly to the cavity, and thoroughly packing with a slightly heated instrument.

On motion the subject was passed.

At the evening session the society elected the following officers:

*President*—Dr. George Elliott, Meadville.

*First Vice-President*—Dr. J. L. Baker, West Chester.

*Second Vice-President*—Dr. J. W. Rhone, Bellefonte.

*Recording Secretary*—Dr. E. Kremer, Lebanon.

*Assistant Recording Secretary*—Dr. W. B. Miller, Altoona.

*Corresponding Secretary*—Dr. W. H. Fundenberg, Pittsburg.

*Treasurer*—Dr. C. R. Jeffries, Wilmington.

*Board of Censors*—Drs. William H. Trueman, C. S. Beck, James Martin, Alonzo Boice, P. K. Filbert.

*State Examining Board* (two elected yearly)—Dr. S. H. Guilford (re-elected), Dr. E. T. Darby.

Cresson was selected as the next place of meeting.

The following standing committees were appointed:

*Executive Committee*—Drs. W. H. Fundenberg, G. L. Robb, P. K. Filbert, E. T. Darby, W. B. Miller.

*Enforcement of Dental Law*—Drs. W. E. Magill, J. W. Rhone, William H. Trueman.

*Publication Committee*—Drs. S. H. Guilford, E. P. Kremer, C. J. Essig, William H. Trueman, William B. Miller.

*Committee on Legislative Action*—Drs W. F. Litch, Wm. B. Miller, M. B. Lowery, G. L. Robb, W. H. Fundenberg.

### THURSDAY MORNING SESSION.

The time was mainly occupied in a general discussion upon Pyorrhœa Alveolaris, Capping Exposed Pulps, and Dental Prosthesis. Nothing especially new was presented.

Adjourned to meet at Cresson the last Tuesday of July, 1885.

NOTES.

Dr. Wingate, of Carbondale, exhibited a number of unique specimens of teeth that had been fractured in the mouth, and had united. All but one were from young patients. The exception was evidently an adult tooth, an incisor, and had been split almost the entire length of the root, and the parts slightly separated. Union had taken place by the same process by which fractured bones are repaired, the space being bridged over, except at one point, where an opening existed communicating with the pulp chamber, and this defect no doubt caused the loss of the tooth. One of the others had attached to it a bony process, starting from about the point of fracture (the fracture had been a transverse one about where the crown and root unite), and extending at right angles to the tooth about a sixteenth of an inch; it then curved abruptly down, and was perhaps a fourth of an inch long. Evidently when the injury took place a fiber or shred of periosteum was torn off, and had produced what the doctor facetiously termed a "pig-tail." He had six or eight in all. They were all so very much distorted in shape that their extraction was a necessity. He was unable to give their history, beyond the fact that their presence becoming a source of irritation they came to him for relief. The doctor has practiced a long time in the mining regions of Pennsylvania, where a large number of lads are employed in the coal mines, and are constantly exposed to accidents of various kinds. While in the coal mine we had an illustration of the risks they ran. While a train of empty cars, such as are used to carry the coal from the distant workings to the shaft, was passing, drawn by mules and going at a rapid rate, a party of lads employed in the mine rolled over the sides and threw themselves alongside the track. The danger of such an acrobatic feat will be understood when we say that there were scarcely eighteen inches between the top of the car and the roof of the mine, and but little more between the side of the car and the wall. They evidently were used to it, as all landed "standing," and enjoyed our surprise at their sudden and unexpected appearance. A very little miscalculation would be likely to cause a severe blow on the face. Probably the doctor was right in considering the specimens he exhibited the result of "skylarking."

Dr. William B. Miller exhibited an arrangement he had devised for mixing amalgam. He had been in the habit of mixing it in his hand, but fearing that a feeling of uneasiness he had in his arm might be due in some way to the mercury, endeavored in the device to imitate as nearly as possible the palm of the hand and a finger. He took a block of rubber, such as stationers sell for erasing lead-pencil marks, and securing it in the lathe, first with a chisel, and then with a cloth wet with chloroform pressed against it while it was revolving rapidly, moulded it into the shape of a small mortar. The edges were then turned until it fitted tightly in a rubber case, such as is used to cover the magnet of a telegraph sounder. To the other end of the case he fitted a flat disk of glass, filling the space between them with plaster. On this he mixes the phosphate and other cements. For a muller, in mixing amalgam, he uses the rubber tip of a lead-pencil. He thinks that the soft rubber takes hold of the amalgam better, mixing it more thoroughly and quickly than a porcelain mortar. His method of using it is as follows: He adds to the fillings (or alloy) enough mercury to make a very soft paste, placing them in the rubber mortar together, and thoroughly unites them, using the rubber tip as a muller. To remove the excess of mercury he uses an amalgam carrier, consisting of a tube one-eighth of an inch in diameter, in which a piston is so arranged that when pressure is made upon the top of the instrument it descends in the tube. When all is ready the tube is filled with the soft amalgam by pressing it into the mortar. It is then rested against the side of the mortar and pressure made upon the piston. This forces out the surplus mercury. The amalgam may be made very hard if desired. It is then carried to the tooth, and pressure upon the piston lands the amalgam in the cavity. The doctor considers this more convenient than the usual way, as he can regulate the quantity of mercury to the requirements of the case.

Fleer & Co., of 1123 Arch Street, Philadelphia, exhibited a mouth glass, so arranged that when the glass is worn out or injured a new one may be inserted in a few moments, by unscrewing a ring which holds it in place. It also permits a plain and magnifying glass being used in the same frame. The cost is three dollars, including five extra glasses. The plain glasses are supplied at a cost of about forty cents each. The idea seems a good one, but we suggest that the size they have adopted, one inch in diameter, is far too large. Half that, or five-eighths of an inch, would be a much more convenient size.

